# Seroprevalence of Cytomegalovirus among Women of Reproductive Age in Iran: A Systematic Review and Meta-Analysis

**Published:** 2019-02

**Authors:** Maedeh SHARGHI, Hadis MUSAVI, Shabnam Malekpour MANSURKHANI, Wesam KOOTI, Masoud BEHZADIFAR, Hadis ASHRAFI-ZADEH, Milad AZAMI, Roonak SHAHOOEI, Hajar KASHEFI, Leila JOUYBARI

**Affiliations:** 1. Student Research Committee, Kurdistan University of Medical Sciences, Sanandaj, Iran; 2. Student Research Committee, Babol University of Medical Sciences, Babol, Iran; 3. Department of Biology, School of Sciences, Shiraz University, Shiraz, Iran; 4. Cellular and Molecular Research Center, Sabzevar University of Medical Sciences, Sabzevar, Iran; 5. Health Management and Economics Research Center, Iran University of Medical Sciences, Tehran, Iran; 6. Student Research Committee, Ahvaz Jundishapur University of Medical Sciences, Ahvaz, Iran; 7. Faculty of Medicine, Ilam University of Medical Sciences, Ilam, Iran; 8. Clinical Care Research Center, Kurdistan University of Medical Sciences, Sanandaj, Iran; 9. Nursing Research Center, Golestan University of Medical Sciences, Gorgan, Iran

**Keywords:** Cytomegalovirus, Pregnancy, Epidemiology, Prevalence, Iran, Systematic review, Meta-analysis

## Abstract

**Background:**

Human cytomegalovirus (CMV) able to cause infection for an entire lifetime. This systematic review and meta-analysis was conducted to determine seroprevalence of CMV among women of reproductive age in Iran.

**Methods:**

English and Persian databases such as Web of Science (WOS), PubMed, Scopus, Cochrane Library, SID, Iran doc, Iran Medex, Magiran, and Medlib were searched (from 2008 to 2017) accurately using the keywords: Cytomegalovirus, Pregnant women or Pregnancy, Epidemiology, Prevalence and Iran.

**Results:**

Results of 15 studies with total samples of 5253 persons from 2008 to 2017 were combined and meta-analyzed. The pooled prevalence rate of IgG among women was estimated 90% (95% CI: 87–93%). The highest prevalence rate of IgG was in Tehran, Rasht, Mashhad and Yasoj, all 100% (95% CI: 100–100%), and the lowest prevalence was in Jahrom 0.62% (95% CI: 53–71%). The overall prevalence rate of IgM among women was estimated at 0.06% (95% CI: 0.03–0.13%). The highest prevalence rate of IgM was in Kerman 0.34% (95% CI: 0.29–0.39%) and Mashhad 0.25% (95% CI: 0.2–0.31%), and the lowest prevalence was in Yasoj 0% (95% CI: 0.00%–0.00%)

**Conclusion:**

The prevalence of immunity in Iran, is satisfactory. Nevertheless, to maintain and increase the level of immunity across the country, it is necessary to routinely screen the women of reproductive ages across the country.

## Introduction

Human cytomegalovirus (HCMV) able to cause infection for an entire lifetime. HCMV is also called human herpesvirus 5 (HHV-5) (1). This virus exists in body fluids and is transmitted from person to person through contact with nasopharyngeal secretions, urine, saliva, semen, cervical secretions, or blood (2). The highest prevalence of this infection has been observed in Africa, Asia, and South America, and the lowest in Western Europe and North America (3). The prevalence of CMV has been reported to be low in Australia, Germany (4) and England (5) while it has been reported to be high in countries such as Saudi Arabia with a prevalence of 90% (6). The prevalence of HCMV antibodies was reported 98% in pregnant women, and 98.3% in non-pregnant women in Brazil (7).

Infection with HCMV is one of the most important causes of death caused by diseases in people with immunodeficiency (8, 9). The infection may be acquired during the embryonic period (congenital) through the placenta as a result of primary infection or recurrent of maternal infection, or during the perinatal/postnatal period, related to cervical secretions, breast milk, or infected blood products (2). HCMV is the most common congenital CMV infection, whose prevalence rates are identical in developed and developing countries (10) and is still considered as a complicated problem for gynecologists and pediatricians (7). Infectious agents during pregnancy, are of particular importance. Because they not only threaten the health of pregnant mothers but also cause fetal death and congenital abnormalities (11). Maternal infection during pregnancy, especially during the first trimester of pregnancy, causes risk of acute fetal infection, and neurological, auditory, and visual disabilities, which will cause a lot of cost and problems (11).

The information about CMV infection in the pregnant women population is very controversial (12). Initial infection with CMV occurs at 0.15% to 2% of all pregnancies and is transferred to the fetus in up to 40% of cases, a number of these cases lead to abortions, about 15% to symptomatic congenital diseases, and 10%–15% to asymptomatic congenital diseases (13). Principally, infections caused by CMV are more common in women, and its risk increases with age (14) but in pregnant women, most of the cases (60%–66%) occur at ages below 30 yr (15).

Given the importance of the health of pregnant women, infants, and children aged 1–59 months (as indicators of social health and development), as well as the serious consequences of CMV for fetus, its screening can be useful during pregnancy (16). Moreover, conducting a comprehensive study to assess the prevalence of CMV in these age groups across the country can be helpful in healthcare planning. The present systematic review and meta-analysis was conducted to determine the prevalence of CMV infection in women of reproductive ages in Iran.

## Methods

### Search strategy

In order to determine the prevalence of CMV infection in reproductive age women in Iran, the current systematic review was designed based on published papers limited to both English and Persian articles from 2008 to 2017. The following English databases were searched: 1) Web of Sciences (WOS); 2) PubMed; 3) Scopus; 4) Embase; and 6) Cochrane Library. The Persian databases were: 1) Magiran; 2) Scientific Information Database (SID), 3) Iran Medex, 4) Iran doc, and 5) Medlib. The databases were searched without time restriction. We searched by the Medical Subject Headings (MeSH) terms such as: “Cytomegalovirus”[MeSH Terms], “Pregnant women”[MeSH Terms], “Pregnancy”[MeSH Terms],“Epidemiology”[MeSH Terms], “Prevalence”[MeSH Terms] and “Iran”[MeSH Terms].

### Inclusion and exclusion criteria

The case-control and cross-sectional studies which determined the prevalence of CMV in both healthy pregnant women and women in their reproductive age following with the serological techniques were included in our systematic review. Inclusion criteria were: Studies which reported rate of CMV antibodies (IgG & IgM) prevalence among an Iranian-based population, case-control and cross-sectional studies, healthy pregnant women, and women in their reproductive age. The exclusion criteria were: studies with non-random sampling methods, those with specific populations (HIV-positive individuals, and immunodeficiency patients), studies with no reports of prevalence or insufficient data, studies which reported cytomegalovirus incidence among non-Iranian nationality, reviews, meeting abstract or editorial comments, and studies having duplicate data.

### Data extraction

In order to assess the eligibility, all relevant papers were reviewed by two independent authors and contradictions among them were dissolved by consensus. The related data were extracted using a data extraction checklist on the basis of title, city, year of publication, author name, sample size, number of seropositive cases (IgG^+^ & IgM^+^) and prevalence of (IgG^+^ & IgM^+^) and diagnostic methods.

The checklists of STROBE for the studies were classified into three categories which included high, intermediate and low quality. Seven items from the recommended checklist of STROBE were selected and used for assessing the quality of studies. The studies were classified as high quality if all items were achieved, as intermediate quality if they did not achieve one criterion, and as low quality if they did not achieve more than one criterion.

### Statistical analysis

A pooled estimated prevalence of cytomegalovirus infection was calculated using Der Simonian-Laird random model (17) and the results were reported by 95% confidence interval (CI). To examine the heterogeneity between studies, Q-test and I2 were used (18). Moreover, to examine possible factors affecting the prevalence, we used relative risk (Relative risk) indicator. To investigate sources of heterogeneity, meta-regression based on the year of publication and sample size were performed. The sensitivity analysis was performed to ensure stability of the results. Studies were ranked according to the year of publication and Cumulative meta-analysis was performed. Subgroup analysis also performed based on geographic location, type of study, and the quality of studies. Egger test and funnel graph were used to evaluate publication bias (19). The data were analyzed using software R ver.3.3.1.

## Results

### Search strategy

**Fig. 1:**
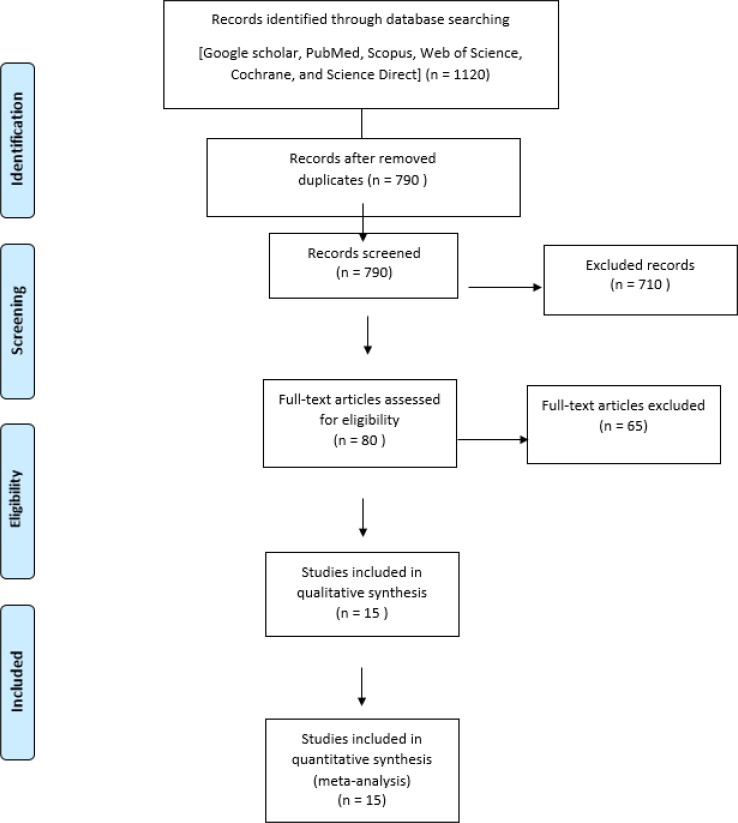
Flowchart describing the study design process

Overall, 1120 articles were identified; 1105 articles were excluded from evaluation due to irrelevant data and duplicated researches. Finally, our meta-analysis study data covers 15 articles from 2007 to 2017 ( Fig. 1).

### General information of samples

Our systematic review cover women of their reproductive age without previous exposure to similar viral infection recently and they had general health.

### Prevalence of cytomegalovirus antibodies among women

Results of 15 studies with total samples of 5253 people from 2008 to 2017 were combined and meta-analyzed. The pooled prevalence rate of IgG among women was estimated 90% (95% CI: 87%–93%) (Fig. 2), sensitivity analysis (Fig. 2-A) and cumulative meta-analysis (Fig. 2-B) were performed but there was no significant change in the overall prevalence.

**Fig. 2:**
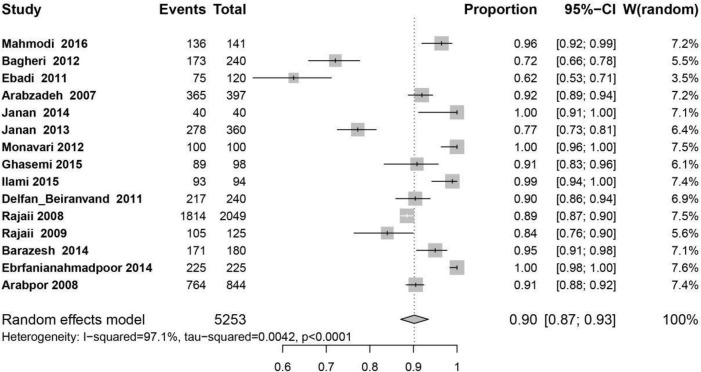
The forest plot of overall prevalence of IgG among women of reproductive age in Iran

**Fig. 2A:**
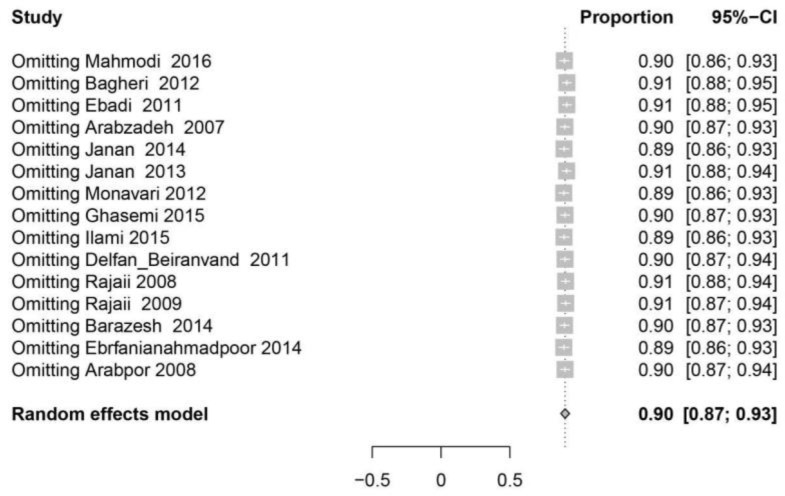
The forest plot of the sensitivity analysis based on overall prevalence of IgG

**Fig. 2B:**
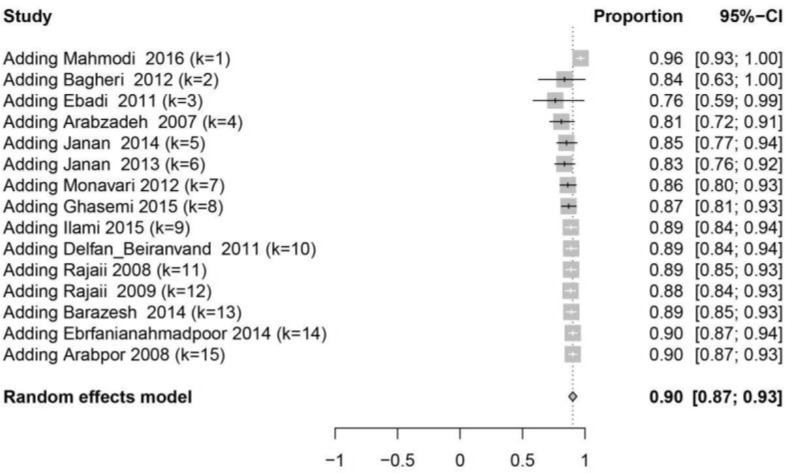
The forest plot of the cumulative meta-analysis based on overall prevalence of IgG

The highest prevalence rate of IgG was in Tehran, Rasht, Mashhad and Yasoj, all 100%, (95% CI: 100%–100%), and the lowest prevalence was in Jahrom 0.62% (95% CI: 53–71%) (Fig. 2) (Table 1).

**Table 1:** Characteristics of all eligible studies

***No.***	***City***	***Year***	***Study population***	***Cytomegalovirus seroprevalence (%)***		***Method***	***Reference***
			(n)	IgG	IgM		
1	Babol	2016	141	0.9645	_	ELISA	(27)
2	Gonabad	2012	240	0.7208	0.25	ELISA	( 28 )
3	Jahrom	2011	120	0.625	_	ELISA	( 29 )
4	Kerman	2007	397	0.9193	0.3375	ELISA	( 11 )
5	Rasht	2014	40	1	0.175	ELISA	( 30 )
6	Rasht	2013	360	0.7722	0.0138	ELISA	( 31 )
7	Tehran	2012	100	1	0.03	ELISA	( 32 )
8	Tehran	2015	98	0.9081	0.1224	ELISA	( 33 )
9	Yasoj	2015	94	0.9893	0	ELISA	( 34 )
10	Khoramabad	2011	240	0.9041	_	ELISA	( 35 )
11	Tabriz	2008	2049	0.8853	0.0003	ELISA	( 36 )
12	Tabriz	2009	125	0.84	0.064	ELISA	( 37 )
13	Boshehr	2014	180	0.95	_	ELISA	( 38 )
14	Mashhad	2014	225	1	0.0266	ELISA	( 39 )
15	Shiraz	2008	844	0.9052	0.0533	ELISA	( 40 )

**Fig. 3:**
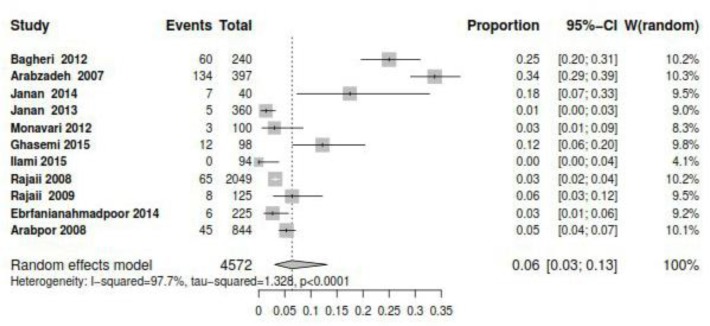
The forest plot of overall prevalence of IgM among women of reproductive age in Iran

The overall prevalence rate of IgM among women was estimated 0.06% (95% CI: 0.03–0.13%). The highest prevalence rate of IgM was in Kerman 0.34% (95% CI: 0.29–0.39%) and Mashhad 0.25 % (95% CI: 0.2%–0.31%), and the lowest prevalence was in Yasoj 0% (95% CI: 0.00%–0.00%) (Fig. 3, Table 1).

Meta-regression result based on year and sample size showed in Table 2. The prevalence of IgG increased by year significantly (*P*=0.031). Moreover, reduction in IgG was seen based on sample size but it was not significant (*P*=0.388).

**Table 2:** Meta-regression result based on year and sample size of studies

***Variable***	***Estimate***	***SE***	***Z***	***P***	***Low CI 95%***	***Upper CI 95%***
Year	0.0110	0.0051	2.1510	0.0315	0.0010	0.0210
Sample size	−0.0000	0.0000	−0.8622	0.3886	−0.0001	0.0000
intercept	−0.0890	0.0187	−4.7581	<.0001	−0.1257	−0.0524

**Fig. 4:**
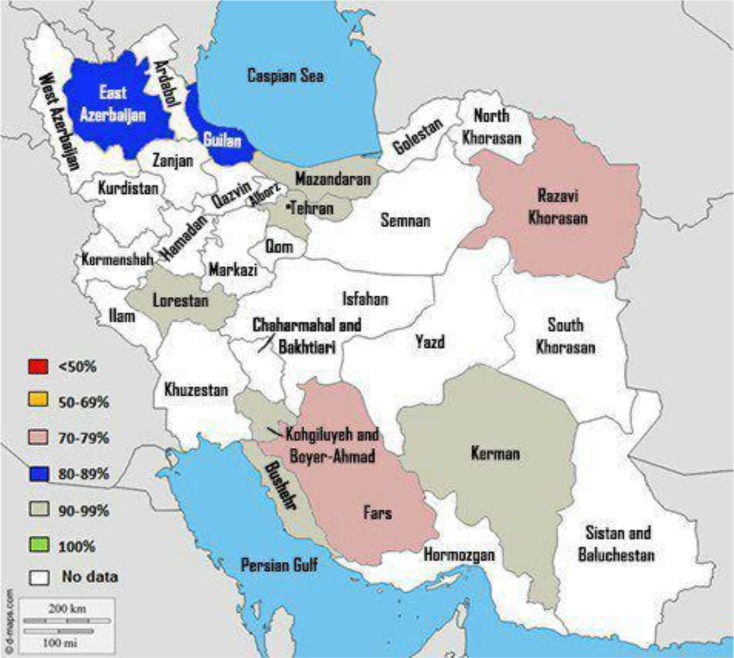
Immunity to cytomegalovirus among women of reproductive age in different regions

Subgroup analysis for comparison of IgG prevalence based on quality of studies, geographical regions, and educational level was shown in Table 3. Among region subgroups, the lowest and highest percentage of positive cases were seen in South 0.85% (95% CI=0.77–0.94%) and Center 0.91% (95% CI=0.87–0.100%) respectively (Table 2 and Fig. 4). Among educational level subgroups, the lowest and highest percentage of positive cases were seen in Under Diploma 0.66 % (95% CI=0.48%–0.90%) and Academic 0.91 (95% CI=0.56%–0.89%), respectively (Table 3).

**Table 3:** Subgroup analysis for comparison of IgG prevalence based on main related factors

***Items***	***No studies***	***Prevalence 95% CI***	***I^2^***	***P***
Quality of studies				
Good	7	88% (82–94)	95.1%	0.0001
Medium	7	91% (86–95)	98.2%	0.0001
Low	1	95% (92–98)	NA	NA
Geographic regions				
North	3	91% (80–100)	96.8%	0.0001
South	3	85% (77–94)	94.2%	0.0001
West	4	91% (85–97)	96%	0.0001
East	3	88% (79–99)	97.9%	0.0001
Center	2	96% (87–100)	88.3%	0.0034
Type of study				
Case-control	3	84% (71–100)	96.1%	0.0001
Cohort	1	91% (89–93)	NA	NA
Cross-sectional	11	91% (87–95)	97.4%	0.0001
Educational level				
Under Diploma	4	66% (48–90)	95.8%	0.0001
Diploma	4	69% (55–86)	90.7%	0.0001
Academic	4	71% (56–89)	78.5%	0.0030

Subgroup analysis of the quality of studies according to the STROB checklist showed that seven studies had Good and Medium quality, and one study was of low quality.

For finding relation between abortion history in women with CMV infection, we used RR indicator, but the result showed no significant relation (RR=0.75 (95% CI: 0.33%–1.70%).

**Fig. 5:**
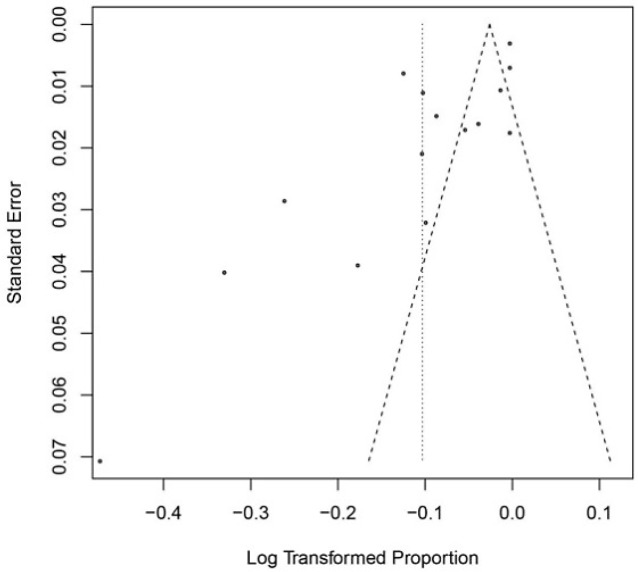
Egger’s regression test to detect publication bias based on overall prevalence of IgG

Funnel plot diagram of IgG antibodies based on Egger’s regression test to detect publication bias is shown in Fig. 5. The results showed publication bias among the studies (*P*=0.0029).

## Discussion

CMV infection in women of reproductive ages causes irreversible complications such as abortions, premature birth, congenital diseases, severe neurological complications, and even death, which subsequently brings a lot of cost and problems to the country. The present study is the first systematic review and meta-analysis, which deals with investigating the prevalence of CMV in women of reproductive ages in Iran.

Overall, based on our meta-analysis, the immunity against cytomegalovirus in women of reproductive ages in Iran was calculated to be 90% (95% CI: 87%–93%) (Fig. 2).

In China, research on 1023 women, the immunity against CMV reported to be 98.7%, which is higher than that in our study (20). In addition, a study in Nigeria on 179 pregnant women, reported the immunity to be 97.2% (21). This level of immunity was also higher than that in Iran. Nevertheless, another study in Sudan on 231 pregnant women and the immunity against CMV was reported to be 72.2% (22) which is lower than that in Iran. In Mexico, the immunity against CMV reported to be 65.6%, which is lower than that in Iran (23).

In our study, the highest level of immunity against CMV was reported to be in Tehran (2012) and Mashhad (2014) with a prevalence of 100%, and the lowest immunity level was related to Jahrom (2011) with a prevalence of 62%. Probably, a higher level of immunity is due to a stronger first-level care, the more efficient and a larger amount of education, the increased level of public awareness, better socio-economic conditions, and a decreased number of pregnancies, and a lower level of immunity due to high-risk sexual behaviors, increased cases of transfusion, decreased family income, as well as decreased levels of education and nursing care.

A sub-group analysis based on geographic regions showed that the highest level of immunity is related to the eastern part (97.9%) and northern part of the country (96.8%), and the lowest level of immunity to the central part of the country (88.3%). Moreover, immunity was calculated to be 96% in the western part of the country, and 94.2% in the southern part of the country. A higher level of immunity is due to the increased level of the people’s education and awareness, as well as a larger amount of education and better socio-economic situation of the people. On the other hand, lack of facilities, increased population, decreased level of informing, decreased levels of social and cultural facilities, decreased level of nursing care quantity and quality, increased amount of high-risk behaviors, and low maternal age at first pregnancy, have resulted in the lowest level of immunity in the central part of the country.

IgM is an antibody, whose titer increases before IgG, with the onset of illness. This antibody is also produced in the stages of reinfection and active infection. In our study, the prevalence of positive IgM was 0.06% in Iran (Fig. 3), whose largest amount belonged to the city of Kerman with 32%, and the lowest to the city of Tabriz with 0.003%. In the United States, the prevalence of CMV was reported to be 1.6% per year, which is greater than that in Iran (24).

A meta-regression based on the year of publication showed that the immunity against CMV has had an increasing trend across Iran during the years 2008 to 2017 (Table 2). This increased level of immunity is due to the improved socioeconomic situation, the increased level of the people’s education and awareness, providing information by the Ministry of Health and it’s paying more attention to first-level care in the country in recent years.

The results of publication bias were significant according to Egger’s test. The quality of the papers affects the bias. The quality of 8 papers out of 15 papers has been at a medium level in our study. Some of the studies have mainly paid attention to pregnant women and married women, which could be a reason why publication bias is significant in our study.

In our study, heterogeneity was calculated using Q-test and I^2^, and its level was reported to be high. One of the reasons for the increased level of heterogeneity can be the small sample size in different studies. In our study, the lowest sample size contained 40 samples, and the highest sample size contained 2,049 samples.

The results showed that the prevalence of CMV was not significantly different between people with a history of abortion and normal group. In a study in Greece on 102 women who had a history of spontaneous abortion of the fetus, there were only 4 people infected with cytomegalovirus (CMV), and no statistically significant relationship was observed between CMV and spontaneous abortion of the fetus (25). In a study conducted on 60 women with a history of abortion, they came to the conclusion that only 9 women (15%) out of those with a history of spontaneous abortion of the fetus, were infected with CMV (26). The results of these studies did not approve the relationship between CMV infection and abortion and were consistent with the results of our study.

Another factor which can be effective in the immunity against CMV is the education level. A subgroup analysis based on the education level showed that the highest level of immunity was related to the education level lower than high school diploma with 95.8%, and the lowest level of immunity was related to academic education with 78.5%. In Sudan, the level of immunity against cytomegalovirus was lower in lower educational levels (22). Moreover, the level of immunity against cytomegalovirus was reported to be higher in people with higher levels of education, which is not consistent with our study (21). Perhaps, these results are due to the small sample size in studies conducted in Iran, as well as the small number of studies which investigated the educational levels.

## Conclusion

Overall, the prevalence of immunity in Iran is satisfactory. Nevertheless, to maintain and increase the level of immunity across the country, it is necessary to routinely screen the women of reproductive ages across the country. In addition, in the provinces where the prevalence of infection has not been measured so far, it should be assessed and reported. Due to its irreversible neonatal complications, CMV testing should become obligatory during pregnancy, to prevent the birth of children with severe neurological problems. The prevalence of CMV infection is higher at early ages, it is necessary to begin teaching how to prevent high-risk sexual behaviors from early ages.

## Ethical considerations

Ethical issues (Including plagiarism, informed consent, misconduct, data fabrication and/or falsification, double publication and/or submission, redundancy, etc.) have been completely observed by the authors.
